# Baseline and extensions approach to information retrieval of complex medical data: Poznan's approach to the bioCADDIE 2016

**DOI:** 10.1093/database/bax103

**Published:** 2018-03-12

**Authors:** Artur Cieslewicz, Jakub Dutkiewicz, Czeslaw Jedrzejek

**Affiliations:** 1 Department of Clinical Pharmacology, Poznan University of Medical Sciences, Dluga 1/2 Str., 61-848 Poznan, Poland; 2 Institute of Control, Robotics and Information Engineering, Poznan University of Technology, ul. Piotrowo 3a, 60-965 Poznań, Poland

## Abstract

Information retrieval from biomedical repositories has become a challenging task because of their increasing size and complexity. To facilitate the research aimed at improving the search for relevant documents, various information retrieval challenges have been launched. In this article, we present the improved medical information retrieval systems designed by Poznan University of Technology and Poznan University of Medical Sciences as a contribution to the bioCADDIE 2016 challenge—a task focusing on information retrieval from a collection of 794 992 datasets generated from 20 biomedical repositories. The system developed by our team utilizes the Terrier 4.2 search platform enhanced by a query expansion method using word embeddings. This approach, after post-challenge modifications and improvements (with particular regard to assigning proper weights for original and expanded terms), allowed us achieving the second best infNDCG measure (0.4539) compared with the challenge results and infAP 0.3978. This demonstrates that proper utilization of word embeddings can be a valuable addition to the information retrieval process. Some analysis is provided on related work involving other bioCADDIE contributions. We discuss the possibility of improving our results by using better word embedding schemes to find candidates for query expansion.

**Database URL**: https://biocaddie.org/benchmark-data

## Introduction

Biomedical research produces ever increasing amount of digital data, which is stored in a variety of formats and hosted in a multitude of different sites. These sites could be generated by original researchers, attached to journals as supplementary material, organized as datasets and kept in databases or repositories. The most common information source is literature in the form of indexed journals that in electronic form reside of Pubmed platform or publisher portals. The article format has its advantage—ease of reading. Articles contain mostly unstructured information that is hard to use specialized processing, comparison, aggregation and integration. Therefore, we need transformation of this information into more structured form that can be stored in databases, collection and repositories. This process requires development of useful data structures and indexing and extraction tools.

Data is a set of values of qualitative or quantitative variables. Pieces of data are individual pieces of information. A dataset or collection of data often corresponds to the contents of a single database table, or a single statistical data matrix, where every column of the table represents a particular variable. Generic ontologies and metadata models designed for description of datasets, supplement domain-specific ontologies to describe the research field. The enormous amount of biomedical literature, the existence of data of different granularity and data heterogeneity, as well as the lack of common metadata, makes it difficult to selectively access increasingly complex relevant information.

As pointed out by (20), ‘A typical dataset available in, for instance, the gene expression repositories may contain a description, a list of keywords and a list of organisms. A typical dataset available in the protein structure repositories contains, in addition, a list of genes and a list of research articles’. Thus, a global pharmaceutical company, for instance, may need close to 30 different databases to complete a clinical study. These sources of data require recording provenance for datasets and data curation. Moreover, the data resulting from biomedical experiments often possess an implicit hierarchy (1). In terms of granularity needed for specific databases, a PubMed article needs to be decomposed into snippets which describe structured data markup. Snippets may be organized using a comprehensive data type ontology which will provide definitions of types of data (Protein, Phenotype, Gene Expression, Nucleotide Sequence, Clinical Trials, Imaging Data, Morphology, Proteomics Data, Physiological Signals, Epigenetic Data, Data from Papers, Omics Data, Survey Data, Cell Signalling and Unspecified). Snippets in different databases may often be found at different levels of a database schema. Since different types of metadata are of importance for given specialized databases, historically their schemas were developed independently, and do not conform to any standardized pattern. Since datasets are combination of structured and unstructured data, often presented in incompatible ways (e.g. the same information with different tags), using them in a complex processing can be quite difficult. Futhermore, a significant percentage of specific data that had been reported in clinical reports does not made its way into journals (2). Nevertheless, data needs to be compared and verified.

Often, cost and utility considerations make it necessary to try a multi-sponsored clinical development approach termed Portfolio of Innovative Platform Engines, Longitudinal Investigations and Novel Effectiveness to generate a new hypothesis. In such environment (3), this need for shared collaborative data governance forces a use of integrated data—therefore, improving the effectiveness of retrieval is paramount to finding state-of-the-art methods of diagnosis, testing and treatment for individual patients. Existing platforms such as Google and PubMed serve their purpose providing an up-to-date sources of information with various additional functionalities but it is difficult to assess their effectiveness. Thus, the crucial aspect for addressing this complexity is the availability of annotated distributed datasets created by the scientific community, with which researchers can test the effectiveness of various approaches. That in turn leads to better data structures and indexes of various granularities. This can be achieved only within a shared task environment, which enables researchers from many different institutions to work together at solving important scientific problems. In the biomedical area, Text REtrieval Conference (TREC) and bioASQ have contributed the most towards achieving this goal. Collaboration occurs at multiple level: definition of test collections, task definition, evaluation and analysis of results. For the last several years, the National Institute of Standards of Technology’s the TREC has concentrated on finding the most relevant PubMed articles and clinical trial data in response to selected medical records within its clinical decision support (CDS) track evolving into Precision Medicine (4). In this context, the bioASQ (5) challenge concentrates mainly on the following broad tasks:

1. bioASQ Task on Online Biomedical Semantic Indexing—classification of new PubMed documents into the MeSH hierarchy concepts.
2. bioASQ Task on Biomedical Semantic query answering (QA) related to information retrieval and query answering—one of the most complex semantic tasks in natural language processing (NLP).

Previous TREC CDS and earlier medical tracks and bioASQ challenges had many specific task orientations, data sources and retrieval conditions. For example, some TREC sources were either full publications or abstracts. The topics of a question could be electronic health records (EHR) admission notes curated by physicians. Notes could be of Diagnosis, Test and Treatment type. Notes could be much longer compared with concise bioCADDIE questions. Currently, the format for run submissions of TREC and bioCADDIE is the standard trec_eval format. The bioASQ contest shares a deep semantic approach to answer questions with bioCADDIE when word embeddings (WEs) are used for query expansion or within document vector framework.

Based on these tasks, the Biomedical and healthCAre Data Discovery Index Ecosystem (bioCADDIE) consortium, funded by the US National Institute of Health Big Data to Knowledge program, aims to empower researchers to find data the most efficient way and expand sources and types of data. These would include opinion on research on non-scientific portals (i.e. conversations about scholarly content) together with monitoring attention surrounding particular work (altmetric).

BioCADDIE (6) has developed DataMed, a search engine prototype of Data Discovery Index (DDI), using the data tag suite (DATS) model to support the DataMed discovery index (7). This enables searching data of various types and formats (while maintaining a core set of elements), curated by separate institutions. DataMed based on ISA formatted metadata aims to facilitate the discovery of a digital object. At this time, DataMed has indexed close to 1 400 000 datasets drawn from 66 repositories (8).

The bioCADDIE challenge concerned finding most relevant docnos (elements of datasets) in response to 15 questions provided by bioCADDIE experts. The structure of the questions followed the DataMed prototype idea of the rdf type of relations between entities (‘data type’ = w, ‘biological process’ = x, ‘species/organism’ = y and ‘phenotype’ = z) (9). The graph structure of a query suggests that if we also transformed documents into graph structure the matching process would be at the level of relations and not keywords.

The aim of the 2016 bioCADDIE Challenge (9) was the retrieval of datasets from a collection that is relevant to the needs of biomedical researchers; the purpose was to facilitate the reutilization of collected data and enable the replication of published results. Such work is the focus of WG4 of the bioCADDIE consortium: Use Cases and Testing Benchmarks. The goal is to develop usability specifications/requirements and appropriate benchmarks with associated testing content for DataMed.

To address this goal sections, later discuss the following aspects:

- The Related work section discusses the content of already published bioCADDIE articles
- The Methodology section presents the methods, algorithms and solutions prepared by our team, divided into following subsections:
  - The Overview, describing the model of our information retrieval system
  - The Collection, with information on the bioCADDIE datasets
  - An Analysis of document structure and content, presenting the differences among various repositories
  - A Selection of documents with valuable data for indexing, with the description of our algorithm evaluating whether a document is worth indexing
  - An Index of data, including information of corpus preparation for indexation
  - Query preprocessing
  - Query expansion, describing the methods chosen to expand the query
  - Information retrieval and evaluation, with information on the retrieval platform
- The Results and discussion section is divided into the following subsections:
- Selection of the optimal baseline system
- Query expansion
- Further analysis
- The Conclusions and future work section summarizes the main outcome of the article

## Related work

At present, details of bioCADDIE Challenge systems exist for selected contributions. Apart from standard similar preprocessing similar to that presented in this work, processing can be divided into advanced preprocessing, retrieval and re-ranking.

The University of California San Diego (UCSD) team that obtained the top infNDCG result (9) implemented a two-step ‘retrieval plus re-ranking’ strategy (10). Based on this idea, they developed a method to find the Google top 10 returned documents and then transformed these documents into queries for relevant datasets. This strategy was used by East China University in their winning contribution to TREC CDS 2015 (11). Their baseline was Elastic search (a Lucene-based search engine that is part of a DataMed technology).

The Elastic search top 5000 retrieved datasets were re-ranked based on the concatenated documents using the pseudo sequential dependence (PSD) model (12). The best run used the PSD-allwords model.

UCSD used the concept matching formula with Dirichlet smoothing, with weights based on the annotated dataset repository. In contrast to the original algorithm in (12), an actual term frequency was increased by a constant = 5. UCSD found (as we do) that neither ordered nor unordered bigrams have improved performance. We would like to point out that the UCSD results presented in (10) do not exactly match the official results (9).

Elsevier (13) used two approaches: word embeddings and ontology-based indexing (queries and data sources were tagged with named entities from MeSH and Entrez Gene) with indexing and search platform Apache Solr. For WEs, fastText (14) gave better results than word2Vec (15) and GloVe (16) that we both used. FastText, based on a skip-gram model, uses character *n*-grams and smaller windows that translate to better WEs for query expansion.

Elsevier used an additional advanced modification of queries:

- Abbreviated species names were expanded to full names (e.g. M to Mus).
- Greek characters were replaced with English spelling.

It has been noted in (13), for example, that for ‘glycolysis’ (a word that does not appear in the bioCADDIE questions), the word2Vec model returned ‘tca_cycle’, ‘mitochondria_remodelling’ and ‘reroute’. FastText delivered more reasonable similar words/phrases. For example, for the phrase ‘glycolysis’, the top three similar phrases returned by fastText were ‘gluconeogenesis’, ‘glycolytic’ and ‘glycolytic_pathway’.

However, it is well-known that WE methods are extremely sensitive to a training corpus (we used the PubMed abstracts). With word2vec, we obtained the following most similar words (characterized by the cosine similarity measure) to ‘glycolysis’: [(gluconeogenesis, 0.804), (glycogenolysis, 0.797), (glyconeogenesis, 0.771), (gluconeogenic, 0.751), (glycogen, 0.7405), (lipogenesis, 0.738), (ureogenesis, 0.738), (glycogenic, 0.738), (ketogenesis, 0.737) and (glycogenolytic, 0.734)].

Elsevier obtained the best result with Elsevier four run modified queries (all additional modifications) + concept expansion + multi-phase execution; Search: Apache Solr, stemmed index) but only 2% better than their baseline.

SIBTex (17) divided query terms into non-relevant, relevant and key, assigning larger weights to key relevant terms compared with relevant terms. This is the same strategy that we used for expanded terms. Universal protein resource (UniProt) was used to constrain query and datasets to a set of 14 biomedical topics (18). They used the Gensim word2vec library (as we did) for finding expansion candidates. Their best run SIBTex 3 was achieved with a baseline + query expansion with weighted terms + results categorization in the post-processing phase.

OHSU assumed a variable number and relative weighting of MeSH terms for query expansion in the work after the challenge. Additional runs determined the optimal number of MeSH terms and weighting. Their best overall score used five MeSH terms with a 1:5 terms: words weighting ratio (19). This is the same ratio we used in our best run when query expanded terms are derived from word2vec.

The University of Melbourne, UM (20) provided useful determination of appearance of most important metadata in bioCADDIE used repositories. This information could be helpful for determination whether a query term belongs to a concept expressed by metadata or using weights for answers coming from different repositories. UM applied transformation of the initial query into a multi-field query that is then enriched with terms that are likely to occur in the relevant datasets.

## Methodology

### The overview

The information retrieval process, we used was divided into four steps:

1. Analysis of repositories structure and their information content.
2. Selection of the optimal baseline system.
3. Selection of optimal possible system extension.
4. Optimization of parameters of the complete system.

The model of the system developed to generate information retrieval for the bioCADDIE challenge includes the following elements:

1. Preparation of database with valuable information from datasets
2. Indexing of data collection
3. Query preprocessing
4. Preparation of two vector space models based on data from bioCADDIE datasets and PubMed abstracts
5. Query expansion with the use of prepared vector space models and pseudo-relevance feedback (PRF) (provided by Terrier)
6. Information retrieval by the Terrier engine
7. Evaluation of the results.

### The collection

The bioCADDIE corpus was a collection of metadata (structured and unstructured) from biomedical datasets generated from a set of 20 individual repositories (Table 1). A total of 794 992 XML documents were made available for use from the set of indices that was frozen from the DataMed backend on 24 March 2016 (21). Data in each document was organized into the following tags:

<DOCNO>: document number,

<TITLE>: document title,

<REPOSITORY>: biomedical repository used to generate document,

<METADATA>: various data from the repository presented in json format.

**Table 1.** Characteristics of the collection

Repository	Description of repository	Number of documents	Number of different json key patterns within <METADATA> tag	Number of documents with valid		
				Title	Keywords	Description
arrayexpress	Data from high-throughput functional genomics experiments	60 881	17	60 817	0	60 804
bioproject	Collection of genomics, functional genomics and genetics studies and links to their resulting datasets	155 850	41	155 631	117 577	149 399
cia	Archive of cancer imaging data	63	1	44	0	63
clinicaltrials	Collection of data concerning publicly- and privately supported clinical studies of human participants conducted around the world	192 500	5518	192 486	138 983	191 934
ctn	Repository of data from National Drug Abuse Treatment Clinical Trials Network	46	1	46	44	46
cvrg	CardioVascular Research Grid	29	5	29	0	28
dataverse	Open-source research data repository software	60 303	7	60 037	0	60 303
dryad	General-purpose database for wide diversity of databases	67 455	98	62 795	60 957	58 421
gemma	Database for genomics data (especially gene expression profiles)	2285	1	2272	0	2285
geo	Datasets focused on gene expression	105 033	4	96 264	0	105 033
mpd	Collection of measured data on laboratory mouse strains and populations	235	1	235	0	235
neuromorpho	Collection of digitally reconstructed neurons associated with peer-reviewed publications	34 082	1	30 016	0	34 082
nursadatasets	Repository of data on the role of nuclear receptors (NRs) in human diseases and conditions in which NRs play an integral role	389	2	389	387	389
openfmri	Collection of magnetic resonance imaging data	36	1	35	0	36
pdb	Database with protein aminoacid sequences	113 493	1410	113 424	113 492	113 331
peptideatlas	Public compendium of peptides identified in mass spectrometry proteomics experiments	76	1	55	0	76
phenodisco	Repository of data from studies investigating the interaction of genotype and phenotype in Humans	429	1	429	0	429
physiobank	The archive containing digital recordings of physiologic signals and related data	70	1	70	0	70
Proteomexchange	Mass spectrometry proteomics data	1716	1	1706	1716	1716
Yped	Open-source proteomics database for high throughput proteomic and small molecule data	21	1	21	0	21
Total		794 992	7113	776 801	433 156	778 701

In many documents, certain data are missing or are removed as uninformative. Of all documents, 97.71% had valid title, 54.49% keywords and 97.95% description. In total, 99.98% had no valid title, keywords or description.

### Analysis of document structures and their information content

Each repository uses different json schema to organize data. Moreover, in some cases a variation was noted within the same repository (Table 1).

To prepare a text corpus for indexing, tags and keys with potentially valuable information were selected and their values were exported to the SQL database. The data was then assigned to one of three categories: Title, Keywords or generalized Description. For one of the repositories (geo), the generalized description contained additional text data, obtained from geo database online resources, based on the ‘geo_accesion’ code found in the metadata (Table 2).

**Table 2.** Preparation of text data for title, keywords and description categories

Repository	Categories		
	Title	Keywords	Description
Arrayexpress	title		description
Bioproject	title	dataItemkeywords	organismtargetspecies, dataItemdescription
Cia	title		anatomicalPartname, diseasename, organismname, organismscientificname
Clinicaltrials	title	keyword	criteria, StudyGroupdescription, Diseasename, Treatmentdescription, Treatmentagent, Datasetdescription
Ctn	title	datasetkeywords	datasetdescription, organismscientificName, organismname
Cvrg	title		datasetdescription
Dataverse	title		publicationdescription, datasetdescription
Dryad	title	datasetkeywords	datasetdescription
Gemma	title		dataItemdescription, organismcommonName
Geo	title		dataItemsource_name, dataItemorganism, dataItemdescription, text data downloaded from geo database on the basis of the geo_accesion code
Mpd	title		datasetdescription, organismscientificName, organismname
Neuromorpho	title		anatomicalPartname, cellname, organismscientificName, organismname
Nursadatasets	title	datasetkeywords	datasetdescription, organismname
Openfmri	title		datasetdescription
Pdb	title	dataItemkeywords	dataItemdescription, organismsourcescientificName, organismhostscientificName, genename
Peptideatlas	title		datasetdescription, treatmentdescription
Phenodisco	title		inexclude, desc, disease, history
Physiobank	title		datasetdescription
Proteomexchange	title	keywords	organismname
Yped	title		datasetdescription, organismname

Items in the table represent column names from the SQL database (prepared on the basis of documents’ JSON keys). In most cases, more than one column was used to prepare text categorized as Description. Thirteen repositories did not provide any keywords.

### Selection of documents with valuable data for indexing

Because documents from some repositories (e.g. dryad, geo) contained very little useful information (see examples in Table 3), we decided to assess if a document’s content is worth indexing using MeSH. MeSH, which stands for ‘Medical Subject Headings’, is a vocabulary thesaurus used by the National Library of Medicine (NLM) to index articles stored in PubMed (22).

**Table 3.** Examples of datasets having very little useful information

No.	Docu-ment number	Repository	Title	Keywords	Description
1	104242	dryad	chr19	NULL	NULL
2	108196	dryad	Chr8	NULL	NULL
3	124757	bioproject	Sobemovirus	NULL	NULL
4	151909	bioproject	Alphaflexiviridae	NULL	NULL
5	500000	geo	A375R_RPL10a_vivo__ Ronly_vem10d_rep2	NULL	melanoma
6	500002	geo	A375_vitro_vehicle_rep3	NULL	melanoma

NULL means that in the source file there was no information that could be categorized as ‘Title’, ‘Keywords’ or ‘Description’.

At this point it does matter whether we use words or lemmatized words, so we chose to remain with the former. Terrier tokenises a query and documents so various word forms are treated exactly the same. In WE methodology various words forms represent different elements of space but when these words became expanded terms only the token form count.

For each category (Title, Keywords or Description) of each record (from the previously prepared SQL database), a score was calculated according to the following heuristic algorithm that removes meaningless records before indexing (i.e. shown in Table 3):

1. Let *X* represent the total number of words in the record.
2. Let *Y1* represent the number of words which are recognized as English words.
3. Let *Y2* represent the number of words which are not recognized as English words (e.g. ‘MIP-2’, ‘CD69’ and ‘LDLR’) but are recognized as MeSH words found in MeSH terms (descriptors and their synonyms in the MeSH database).
4. If there are no words (e.g. the document is lacking keywords) set the Score to −1.
5. Calculate the Score = (*Y1* + *Y2*)/*X*.
6. For Title and Description categories, if the Score is >0 and *X* is >2, take the record for indexing.
7. For the Keywords category, if the Score is >0 (as follows from the condition at Step 4), take the record for indexing.

The Descriptor/Concept/Term structure makes it possible to attach various data elements in MeSH to the appropriate object. This sentence is directly taken from https://www.nlm.nih.gov/mesh/concept_structure.html. Word (linguistic notion), term (appears in a query), data element (part of a taxonomy structure) differ in context—here they are used in the meaning of word.

MeSH terms used in the previous algorithm were prepared according to the following procedure:

1. ‘Descriptor’, ‘Substances with pharmacologic action’, ‘Qualifiers’ and ‘Supplementary records’ files were downloaded from the MeSH database website (20).
2. Words were collected from specific tags depending on the file (<DescriptorName> and <ConceptList> tree from ‘desc2017.xml’; <DescriptorName> and <PharmacologicalActionSubstanceList> tree from ‘pa2017.xml’; <QualifierName> and <ConceptList> tree from ‘qual2017.xml’; <SupplementalRecordName> and <ConceptList> tree from ‘suppl2017.xml’).
3. For each word characters such as ‘,’, “(‘or’)” were removed.
4. The list of words was reduced by removing duplicates of each word.
5. The resulting list of unique terms consisted of 479 545 words.

### Indexing of data

After the removal of documents without valuable data, the text corpus for indexing was prepared in the form of an xml file, with the content of every document placed within a DOC tag (a format required by Terrier). Such prepared text corpora were tokenized and indexed by the Terrier 4.2 engine (8).

### Query preprocessing

The queries were provided as natural language sentences, containing a lot of noise words. To improve the retrieval, stop-words and common non-informative phrases (e.g. ‘find’, ‘data’ and ‘related to’) were removed from each query.

### Query expansion

To expand the queries, we used WEs, choosing the word2vec algorithm (15). Two vector space models were calculated the first based on the corpus from the bioCADDIE collection and the second utilizing the much larger text corpus based on PubMed article abstracts. Calculated vectors were then used to find the words most similar to query terms. To enable setting the different weights for original and expanded query terms, the query was not passed through the tokenizer (class SingleLineTRECQuery).

Additional query expansion was carried out by the Terrier engine in the form of PRF utilizing the Rocchio algorithm.

### Information retrieval and evaluation

Information retrieval was done using the Terrier 4.2 platform. The results were then evaluated using the qrel file provided by the challenge organizers.

## Results and discussion

The complexity and fragmentation of the repositories made it difficult to index the data. For the original challenge, due to lack of time and inexperience of our team with DataMed, the data was not fully indexed and we achieved a poor result, shown in Table 4 (9).

**Table 4.** Original Poznan consortium results as submitted for the challenge vs. the best participant results for a given evaluation measure (in bold font)

Group	Submission	infAP	infNDCG	NDCG@10	P@10 (+partial)	P@10 (−partial)
IAII_PUT	Biocaddie dphresults.txt	0.0876	0.3580	0.4265	0.5333	0.1600
UCSD	armyofucsdgrads-3.txt	0.1468	**0.5132**	0.5303	0.7133	0.2400
SIBTex	sibtex-5_0.txt	**0.3664**	0.4188	0.6271	0.7533	0.3467
Elsevier	elsevier4.txt	0.3049	0.4368	**0.6861**	**0.8267**	**0.4267**
UIUC GSIS	sdm-0.75-0.1-0.15.krovetz.txt	0.3228	0.4502	0.5569	0.7133	0.2867
BioMelb	Post-challenge	0.3575	0.4219		0.7733	
*Poznan—this work*	*LGD word2vec and Terrier Rocchio*	***0.3978***	*0.4539*	*0.6375*	*0.7700*	*0.4000*

The results of the current Poznan consortium work are shown in italics.

Having made modifications of our system, our present results are much better. Application of our algorithm for selection of documents with valuable data for the indexing revealed that 97.71% of documents had ‘Title’ assessed as valid for indexing (see Table 1 for details). A similar value was observed for ‘Description’ (97.95%). Only slightly more than half of documents (54.49%) had valid keywords (this was mainly due to the fact that in many datasets keywords were not present). One hundred and fifty-five datasets were assessed as having no valid ‘Title’, ‘Keywords’ and ‘Description’. Only one of them was present in the qrels file (dataset no. 5322) and was marked as ‘non-judged’ (−1).

### Selection of the optimal baseline system

Our selection of Terrier (23)—the open-source search engine written in Java—was motivated by its maturity and its use of state-of-the-art retrieval weighting models and techniques that can be used to index large collection of various documents.

In particular, some of the notable weighting models implemented include Okapi BM25 (best matching model), term frequency inversed document frequency (TFIDF) and a whole group of Divergence From Randomness Framework, DFR [mostly originating in (24)]. DFR models have their origin in information theory (Amati, Encyclopedia). A word that is randomly distributed according to some distribution in documents is not informative, whereas a word that does not obey this distribution conveys information. The models were obtained by representing the three components of the framework: selecting a basic randomness model, applying the first normalization and normalizing the term frequencies with respect to the document-length. In this work, the so-called Normalization 2 was applied with the hyper-parameter *c* = 1.

The following divergence from randomness (DFR) models were used based on Terrier (DFR Framework, http://terrier.org/docs/v3.5/dfr_description.html):

BB2 (Bernoulli–Einstein model with Bernoulli after-effect and Normalization 2),

DFR version of BM25, DFree (parameter-free DFR model),

DLH and its improved version DLH13 (parameter-free DFR model, assuming hypergeometric term frequency distribution),

DPH (parameter-free hypergeometric model with Popper’s normalization),

IFB2 (inverse term frequency model with Bernoulli after-effect and Normalization 2),

ExpB2 (inverse expected document frequency model with Bernoulli after-effect and Normalization 2; it uses logarithm Base 2),

In_ExpC2 (same as the previous one but with logarithm base e),

InL2 (inverse document frequency model with Laplace after-effect and Normalization 2),

LGD (a log-logistic model for information retrieval) (23, 25) and

PL2 (Poisson model with Laplace after-effect and Normalization 2).

We direct a reader to the original source (26) for complex model formulas. So far, it has not been demonstrated theoretically why some of these models perform better than others.

Another valuable feature implemented in Terrier is PRF query expansion—a mechanism allowing for extraction of *n* most informative terms from m top ranked documents (ranking created in the first search run) which are then added to the original query in the second retrieval rank. Terrier provides both parameter-free (Bose–Einstein 1; Bose–Einstein 2; Kullback–Leibler) and parameterized (Rocchio) models for query expansion (27). The Rocchio feedback approach was developed using the vector space model. The modified vectors are moved in a direction closer or farther away, from the original query depending on whether documents, are related or non-related.

In recent work (28), several leading systems were evaluated within the Open-Source Information Retrieval (IR) Reproducibility Challenge for the Gov2 test collection to select the best DFR variant. Among the options was Terrier 4.0 with DPH ranking function, which is a hypergeometric parameter-free model from the Divergence from Randomness family of functions (8). The query expansion version—the ‘DPH  +  Bo1 QE’ uses PRF, which is known to find potentially relevant terms by first querying the index and looking for new terms in high-ranking documents. Specifically, 10 terms are added from three PRF documents.

Research by in (28) found that the ‘DPH  +  Bo1 QE’ run of Terrier 4.0 was statistically significantly better than all other runs including Terrier’s BM25 run, with all other differences not significant. In particular, it was 0.04 better compared with the Lucene-based solutions for the mean average precision (MAP) at 1000 measure. We corroborated this finding with the relatively successful Poznan University of Technology (PUT) TREC CDS 2016 contribution (29), where Terrier DPH Bo1 was used, and the data consisted of a subset of the PubMed articles.

The baseline information retrieval results are presented in Table 5. Fourteen weighting models implemented in Terrier were tested, with the log-logistic DFR model providing the best infNDCG.

**Table 5.** Baseline information retrieval results

Algorithm	infAP	infNDCG	P@10 (+partial)	P@10 (−partial)
BB2	0.3550	0.4184	0.7133	0.3533
BM25	0.3547	0.4055	0.7067	0.3400
DFR_BM25	0.3723	0.4085	0.7067	0.3533
Dfree	0.3664	0.4248	**0.7533**	**0.4067**
DLH	0.3617	0.4120	0.7200	0.3333
DLH13	0.3640	0.4207	0.7533	0.3733
DPH	0.3442	0.4125	0.7200	0.3400
IFB2	0.3494	0.3948	0.6853	0.3400
In_ExpB2	0.3534	0.4079	0.7222	0.3667
In_ExpC2	0.3379	0.4015	0.7367	0.3333
InL2	**0.3791**	0.4181	0.7367	0.3600
LGD	0.3773	**0.4355**	0.7333	0.3933
PL2	0.3474	0.4009	0.7222	0.3067
TFIDF	0.3530	0.4120	0.7067	0.3400

Bold font indicates the highest values for a given measure.

For the Biocaddie data, which are not continuous data, surprisingly the best results for infNDCG were achieved with LGD, not BB2 (DPH Bo1), which provides the best results for infAP and P@10. These results could not had been predicted before the evaluation of the Challenge results. Therefore, for original challenge our results could have been 0.02 lower in comparison to what we present now.

Our baseline results compare quite favourably with the best original baseline bioCADDIE teams’ results in spite of the fact that no advanced preprocessing was used. The best Terrier option LGD gives the infNDCG value 0.4355, compared with UCSD 0.4498 (official bioCADDIE evaluation)/0.433 (10), and Elsevier’ 0.4292 (13), UIUC GSIS 0.4207, SIBTex 0.3898 (17).

### Query expansion

Expanding queries by adding potentially relevant terms is a common practice in improving relevance in IR systems. There are many methods of query expansion. Relevance feedback takes the documents on top of a ranking list and adds terms appearing in these document to a new query. In this work, we use the idea to add synonyms and other similar terms to query terms before the PRF. This type of expansion can be divided into two categories. The first category involves the use of ontologies or lexicons (relational knowledge). In biomedical area UMLS, MeSH (22), SNOMED-CT, ICD-10, WordNet and Wikipedia are used (30). Generally, the result of lexicon type expansion is positive (in the bioCADDIE contest see for example (19, 20)). We did not use this method in our work because of lack of access to MeSH medical text indexer service. The second category is WE, i.e. word2vec—mapping a word on a corresponding vector. This belongs to a class of distributional semantics, feature learning techniques in natural language processing. Such language modelling derives word space from linguistic items in context. Space with one dimension per word is transformed to a continuous vector space with much lower dimension. Meaning is obtained by defining a distance measure between vectors corresponding to lexical entities (here words). In the WE query expansion methods, terms are added to a query based on their similarity to original query terms. Goodwin and Harabagiu (31) used the skip-gram word2vec method for query expansion with negative effect compared with the baseline, as we did for TREC CDS (29).

Analysis of the effects of query expansion is difficult, as stressed in (32). There, it was shown that various methods gave very different top expansion terms in response to a query ‘foreign minorities Germany in Google (as of April 2009)’. The methods were automatic-query expansion, mutual information, local context analysis Rocchio, binary independent model, Chi-square, Robertson selection value, Kullback–Leibler and relevance model. Only the binary independent model, Chi-square and Kullback–Leibler gave ‘frisians’ and sorbs ‘2’ as the top two expanded terms. Some of the methods got none of the intended correct terms among the first eight expanded terms.

In this work, we used MeSH only for filtering, so that query expansion terms stayed in the medical domain. The query was expanded with most similar terms obtained from a collection of PubMed Biomedical journal citations (titles and abstracts) and from the Biocaddie data challenge collection. Similarity was calculated for each dataset using word2vec, an efficient model allowing for learning vector representations of words from unstructured text data (15) with the following parameters:

- PubMed collection: number of dimensions = 100; window size = 5; minimum word count = 10; this resulted in the collection of 1 498 219 words;
- BioCaddie collection: number of dimensions = 100; window size = 20; minimum word count = 5; this resulted in the collection of 296 503 words.

A similarity threshold was set to 0.9 for vectors generated from PubMed abstracts and 0.8 for vectors calculated on the basis of bioCADDIE datasets (lower values resulted in dissimilar query terms).

As in (29) and (31), if queries are expanded with WE obtained terms and added to a list of query terms with the same weight as the original terms, the results, in general, get worse, because a query drift is introduced. In Question 9 (question pertains to ‘ob’ and *Mus musculus*), adding terms such as ‘mouse’ or ‘mice’ to a question does not improve the result.

The most important result of this work is observation that the results improve if query expanded terms are given a much smaller weight than the original terms.

The weight of original query terms was set to 100, terms obtained from PubMed to 20 and terms provided with bioCADDIE embeddings to 1. This is justified by the relative smallness of the bioCADDIE dataset.

In (26), we used MeSH not only for filtering but also for query expansion, with positive results. For the purpose of this work, we use MeSH only for filtering because the free access interface was discontinued.

We tried query expansion with WE using two approaches:

1. The skip-gram method (15) on abstracts of the entire PubMed using Gensim library (33).
2. The Glove method (16) on free TREC 2016 PubMed documents.

In our case, vectors obtained from word2vec and Glove were quite different, and in case of Glove gave negative results (data not shown). However, this may be related to the relative smallness of the corpora used. We plan to extend the current work to larger corpora (e.g. 34) for neural network training.

We focused on the Terrier Rocchio method optimizing the beta parameter, a number of top documents and a number of extracted terms to obtain an optimal infNDCG result. For the same conditions, the Rocchio query expansion method slightly outperforms the Terrier parameter-free expansion method Bo1 http://terrier.org/docs/v3.5/javadoc/org/terrier/matching/models/queryexpansion/Bo1.html). For LGD with word2vec, the difference is 0.0049. For infAP the reverse occurs—the parameter-free expansion slightly outperforms Rocchio by 0.0034.

Terrier PRF was configured to use the Rocchio algorithm with the following parameters: number of top documents used for query expansion = 2; number of terms extracted from each document = 2; beta parameter for Rocchio algorithm = 0.5.

The results of information retrieval with expanded query are presented in Table 6. Once again, LGD was found to provide the best infNDCG measure. The percentage-wise gain obtained by the query expansion over the baseline result is a little over 4%, smaller than achieved in (29). However, the bioCADDIE data have quite irregular structure (some data types missing in many documents), and this might make a difference.

**Table 6.** Baseline information retrieval results with the best word2vec query expansion and PRF

Algorithm	Run parameters	infAP	indNDCG	P@10 (+partial)	P@10 (−partial)
BB2	terrier Rocchio	0.3911	0.4325	**0.7900**	0.3200
BB2	word2vec and terrier Rocchio	**0.4001**	0.4533	**0.7900**	0.3200
BM25	terrier Rocchio	0.3719	0.4158	0.7067	0.3200
BM25	word2vec and terrier Rocchio	0.3601	0.4286	0.6933	0.3200
DFR_BM25	terrier Rocchio	0.3883	0.4066	0.7214	0.3133
DFR_BM25	word2vec and terrier Rocchio	0.3801	0.4311	0.7267	0.3133
Dfree	terrier Rocchio	0.3910	0.4371	0.7500	0.3667
Dfree	word2vec and terrier Rocchio	0.3888	0.4454	0.7567	0.3733
DLH	terrier Rocchio	0.3683	0.4181	0.7400	0.3000
DLH	word2vec and terrier Rocchio	0.3604	0.4292	0.7400	0.3000
DLH13	terrier Rocchio	0.3759	0.4324	0.7733	0.3467
DLH13	word2vec and terrier Rocchio	0.3692	0.4422	0.7733	0.3467
DPH	terrier Rocchio	0.3779	0.4194	0.7500	0.3133
DPH	word2vec and terrier Rocchio	0.3751	0.4276	0.7567	0.3200
IFB2	terrier Rocchio	0.3669	0.4005	0.7233	0.3133
IFB2	word2vec and terrier Rocchio	0.3813	0.4284	0.7367	0.3067
In_ExpB2	terrier Rocchio	0.3720	0.4108	0.7433	0.3133
In_ExpB2	word2vec and terrier Rocchio	0.3816	0.4330	0.7433	0.3133
In_ExpC2	terrier Rocchio	0.3720	0.3999	0.7367	0.3133
In_ExpC2	word2vec and terrier Rocchio	0.3672	0.4157	0.7367	0.3133
InL2	terrier Rocchio	**0.4001**	0.4259	0.7533	0.3133
InL2	word2vec and terrier Rocchio	0.3902	0.4360	0.7467	0.3200
LGD	terrier Rocchio	0.3990	0.4456	0.7633	0.3867
LGD	word2vec and terrier Rocchio	0.3978	**0.4539**	0.7700	**0.3933**
PL2	terrier Rocchio	0.3648	0.4082	0.7467	0.2800
PL2	word2vec and terrier Rocchio	0.3542	0.4213	0.7467	0.2800
TFIDF	terrier Rocchio	0.3641	0.4023	0.7317	0.3133
TFIDF	word2vec and terrier Rocchio	0.3523	0.4154	0.7250	0.3133

Bold font indicates the highest values for a given measure.

### Further analysis

To better understand the results, we did evaluation for individual questions (Table 7) for our best result: LGD with query expanded with word2vec and Terrier PRF. Strikingly, the highest value of measure is for Question 15 (for which, similar to Question 7 no Score 2 of evaluation was assigned).

**Table 7.** Variation of measures for each bioCADDIE question

Query number	infAP	infNDCG	P@10 (partial)
1	0.4217	0.6504	0.9000
2	0.3933	0.3338	0.8000
3	0.5832	0.6898	0.9000
4	0.6999	0.5177	1.0000
5	0.1620	0.2897	0.4000
6	0.3256	0.4938	1.0000
7	0.1931	0.6197	0.2500
8	0.0856	0.4547	0.3000
9	0.2207	0.2607	0.8000
10	0.1186	0.1961	0.5000
11	0.6373	0.3402	1.0000
12	0.5860	0.4011	0.9000
13	0.3171	0.2919	0.9000
14	0.7005	0.3300	0.9000
15	0.5228	0.9384	1.0000
Average	0.3978	0.4539	0.7700

Further analysis of which particular databases carry information gain is required. For example, neuromorpho provided 11% of the contribution to infNDCG measure, although it constitutes <5% of data volume.

Table 8 presents the details of run options for the LGD algorithm using the same or different weights for original and expanded terms and shows that expansion terms should not have the same weight as original terms.

**Table 8.** Evaluation of search results obtained with the LGD algorithm using the same or different weights for original and expanded terms

Run	infAP	infNDCG	NDCG@10	P@10 (+partial)	P@10 (−partial)
Separate words; terms added manually; same weight of all terms	0.2896	0.3329		0.6656	
Separate words; terms added manually; original query words weight = 100	0.3922	0.4525		0.7633	
Terms from query as separate words without query expansion	0.3773	0.4355	0.6375	0.7333	0.4000
Terms from query as separate words; Terrier query expansion (PRF)	0.3990	0.4456	0.6425	0.7633	0.3867
Terms from query (weight 100) + word2vec (weight 20 or 1, depending on the corpus − PubMed or bioCADDIE) + Terrier query expansion (PRF)	0.3978	0.4539	0.6425	0.7700	0.3933

Manually added terms were chosen by a biology specialist.

We evaluated the results using the query relevance file with partially relevant documents denoted as non-relevant. We have noticed that search results benefit from query expansion in any form. We have evaluated three forms of expanding the query: no expansion (denoted as NoEXP), Terrier default query expansion (denoted as Terrier) and query expansion with the WEs (denoted as Emb). Results are presented in Table 9.

**Table 9.** Evaluation of search results obtained with various algorithms without use of partially relevant documents

Expansion method	NoEXP	Terrier	Emb	NoEXP	Terrier	Emb
baseline method	infAP	infAP	infAP	infNDCG	infNDCG	infNDCG
InL2c	0.1940	**0.2056**	0.2085	0.2524	**0.2689**	**0.2687**
BB2	0.1853	0.2023	0.2079	0.2469	0.2624	0.2642
BM25	0.1813	0.1950	0.1980	0.2437	0.2591	0.2610
DFR_BM25	0.1893	0.1996	0.2040	0.2469	0.2590	0.2601
In_expB2	0.1841	0.1954	0.1995	0.2439	0.2578	0.2587
DLH13	0.1780	0.1815	0.1845	0.2495	0.2529	0.2585
LGD	**0.1946**	0.2013	**0.2086**	**0.2599**	0.2569	0.2579
DLH	0.1633	0.1664	0.1688	0.2392	0.2560	0.2579
DFRee	0.1779	0.1905	0.1981	0.2489	0.2547	0.2560
IFB	0.1684	0.1762	0.1824	0.2360	0.2467	0.2485
In_expC2	0.1754	0.1786	0.1829	0.2350	0.2408	0.2420
PL2	0.1660	0.1708	0.1735	0.2367	0.2371	0.2383
DPH	0.1584	0.1691	0.1777	0.2422	0.2343	0.2360
TF_IDF	0.1827	0.1880	0.1904	0.2456	0.2327	0.2345

Bold font indicates the highest values for a given measure.

We can see that commonly used BM25 and its extension InL2 gives surprisingly good results, better than the best performing algorithm in the full evaluation—LGD. In terms of cumulative gain, TF-IDF is the worst performing algorithm. Improvement for results obtained with query expansion is consistent across all algorithms. Composition of both types of query expansions gives the best results, reaching a normalized discounted cumulative gain of 0.2687 for the InL2 algorithm and 0.2086 Average Precision for the LGD algorithm.

## Conclusions and future work

Shared tasks bioCADDIE challenge fulfilled an important role in the advancement of biomedical Information retrieval methods using data snippets as datasets. Our post-challenge analysis indicates that bioCADDIE data is quite different from continuous biomedical data. There are quite a number of documents that basically present the same information duplicated in NML databases. Manual expansion, in general, makes the results worse. Word2vec based query expansion improves the results but expansion term weights have to be much smaller than the original weights. For effectiveness of word2vec, a method for calculating the similarity of candidate expansion terms to the original query terms is crucial. In this work, we use the pure word2vec.

Several recently proposed theoretical approaches to query expansion reporting positive results (34–42) deserve to be applied in a bioCADDIE context [including word2vec (38)]. There are many studies on WE information retrieval in the biomedical domain (34, 39).

The work of Fudan group within the bioASK contest (43) used deep semantics comparing query and document text on a sentence basis (D2V, document vectors). D2V-TFIDF, which concatenates both dense and sparse semantic representations, performed very well in application to ranking of MeSHLabeler.

It should be stressed that in (15), the pure word2vec method (with cosine similarity) was presented as better than it actually is by choosing an easy type of corpus such as countries and capitals. Much better results are obtained when sense disambiguation (44) and hubness reduction is applied to the vector space. For similarity tasks, the results in (45), where three different corrections to word2vec were used (retrofit, hubness removal and ranking type similarity), are up to 30% better than with the other method (15). Such a method (enhanced to relatedness) could allow direct comparison of query and target terms.

Other query expansion schemes are based on WE exist (41–42). Terrier provides a state-of-the-art baseline system but our perspective is that PRF and phrase query expansion could be significantly improved within Terrier.

Direct comparison of this work results with original bioCADDIE results is not warranted. Nevertheless, our results are strong. They are close to the top in most measures, and the best in infAP measure.

To summarize, the main conclusions of this article are the following:

1. Use of language models created on the basis of distribution semantics to expand the query (using WE) has the potential to significantly improve WE results in the near future.
2. Assigning different weights to words in a query, depending on whether the words were added in the expansion process or originating from the original content of the query significantly improves the result.
3. Filtering documents that do not convey informative content (based on PyEnchant and MeSH) likewise improves the result.
4. An important element influencing the final result was the selection of the appropriate ranking function and the adjustment of the PRF extension parameters (parametric, with the coefficient β, to use the two best articles, instead of the standard three).

In achieving the competitive results of this work, we used no advanced preprocessing, neither manual tasks nor system training. These results could be treated as a new baseline. It is our belief that with more sophistication by including the aforementioned elements, particularly in application to individual questions, we can potentially improve infNDCG by 0.05. Even small improvement amounts to a large economic gain as in the 2012 survey (46), it had been found that that doctors performed an average of six professional searches a day during their course of work.

The bioCADDIE challenge results need to be further analysed to understand which features of participating team algorithms contributed to effectiveness of results for particular measures. Such extended analysis was performed or TREC CDS 2014 (47).

Comparing all bioCADDIE runs based on the infAP, infNDCG, NDCG and P@10 there is surprisingly little correlation between evaluated results for these measures (20). The UCSD team was ranked first in term of infNDCG but would rank ninth in the ranking based on the classic NDCG metric. The UCSD method was optimized for infNDCG but has not been universally strong across measures. This challenge deserves further work and should contribute the development of a DDI prototype.

Finally, the result of bioCADDIE effort could be useful for determination of relevance of particular data. For example, evaluation performed in (48) showed that the genome-wide association studies dataset finder outperformed PubMed significantly in retrieving literature with desired datasets. This could indicate better usefulness of datasets compared with literature for some semantic tasks.

## Funding

Poznan University of Technology grant (04/45/DSPB/0149). The bioCADDIE Dataset Retrieval Challenge was supported by National Institutes of Health (U24AI117966).

*Conflict of interest*. None declared.
